# Early integrated palliative care in chronic heart failure and chronic obstructive pulmonary disease: protocol of a feasibility before-after intervention study

**DOI:** 10.1186/s40814-019-0420-y

**Published:** 2019-02-21

**Authors:** N. Siouta, A. Heylen, B. Aertgeerts, P. Clement, J. Van Cleemput, W. Janssens, J. Menten

**Affiliations:** 1 Laboratory of Experimental Radiotherapy-Palliative Care, KU Leuven, Herestraat 49, 3000 Leuven, Belgium; 2 Palliative Support Team, UZ Leuven, Herestraat 49, 3000 Leuven, Belgium; 3 Academic Center for General Practice, KU Leuven, Kapucijnenvoer 33, 3000 Leuven, Belgium; 4 Department of Oncology, KU Leuven, Herestraat 49, 3000 Leuven, Belgium; 5 Department of Cardiology, KU Leuven, Herestraat 49, 3000 Leuven, Belgium; 6 Department of Pneumology, KU Leuven, Herestraat 49, 3000 Leuven, Belgium

## Abstract

**Background:**

Patients with chronic heart failure (CHF) and patients with chronic obstructive pulmonary disease (COPD) are amenable to integrated palliative care (PC); however, despite the recommendation by various healthcare organizations, these patients have limited access to integrated PC services. In this study, we present the protocol of a feasibility prospective study that aims to explore if an “early integrated PC” intervention can be performed in an acute setting (cardiology and pulmonology wards) and whether it will have an effect on (i) the satisfaction of care and (ii) the quality of life and the level of symptom control of CHF/COPD patients and their informal caregivers.

**Methods:**

A before-after intervention study with three phases, (i) baseline phase where the control group receives standard care, (ii) training phase where the personnel is trained on the application of the intervention, and (iii) intervention phase where the intervention is applied, will be carried out in cardiology and pulmonology wards in the University Hospital Leuven for patients with advanced CHF/COPD and their informal caregivers. Eligible patients (both control and intervention group) and their informal caregivers will be asked to complete the Palliative Outcome Scale, the CANHELP Lite, and the Advance Care Planning Questionnaire at the inclusion moment and 3 months after hospital discharge.

**Discussion:**

The present study will assess the feasibility of carrying out PC-focused studies in acute wards for CHF/COPD patients and draw lessons for the further integration of PC alongside standard treatment. Further, it will measure the quality of life and quality of care of patients and thus shed light on the care needs of this population. Finally, it will evaluate the potential efficacy of the “early integrated palliative care” by comparing against existing practices.

**Trial registration:**

Current Controlled Trials ISRCTN24796028 (date of registration August 30, 2018).

## Background

According to the World Health Organization (WHO), chronic heart failure (CHF) is the first cause of death worldwide while chronic obstructive pulmonary disease (COPD) is the third one [1]. Patients with CHF/COPD face heavy physical and psychosocial burdens, comparable to cancer patients [2, 3], and whereas treatment for both diseases remains non-curative, survival rates of these patients have increased with time [4]. These patients are amenable to and can benefit from palliative care (PC) services; however, the complexity of their needs requires a more integrated approach to the provision of high-quality care [4].

Integrated PC involves bringing together administrative, organizational, clinical, and service aspects in order to achieve continuity of care between all those involved in the patient’s care network. It aims to achieve quality of life and a well-supported dying process for the patient and the family in collaboration with all the caregivers (paid and unpaid) [5, 6]. Moreover, there is ample and ever-growing empirical evidence on the benefits of integrated PC on the quality of life of patients with both malignant and non-malignant diseases with typical examples including better symptom control, less caregiver burden, fewer hospital admissions, improvement in continuity and coordination of care, cost-effectiveness, and patients dying in their preferred place [4, 7–15].

However, when compared to patients with cancer, patients with CHF/COPD are quite unlikely to receive PC services and it has been estimated that less than one out of five CHF/COPD patients has access to PC services, while for cancer, this number increases to one out of two [10, 16, 17]. The most commonly reported factors responsible for this inequity are limited PC knowledge of healthcare providers and predominant focus on standard treatment options [18–21], misperception of PC as an end-of-life care [15], complexity of prognostication [18–21], perception of CHF and COPD as “manageable” chronic diseases [22–24], and inadequate communication and collaboration between the involved medical disciplines [6, 25–27].

The nature of these inhibiting factors suggests that optimal practices will combine optimized strategies for the delivery of high-quality care vis-a-vis the efficient and effective organization of services within the local context and societal impact including optimal patient/professional experiences.

## Aim

The aim of this study is twofold: (1) to assess the feasibility of administering an early PC intervention in cardiology and pulmonology wards and (2) to measure the potential benefits of an early PC intervention in CHF/COPD patients in their QoL and satisfaction of care. Additionally, the objectives of this study are as follows:

i) To identify barriers in administering an early integrated PC intervention in an acute setting
ii) To examine the appropriateness of the eligibility (referral) criteria
iii) To measure whether and to what extent patients and their informal caregivers share common beliefs on QoL and quality of care
iv) To test the hypothesis that early integration of PC in CHF/COPD can improve QoL and quality of care
v) To identify the PC needs of the targeted population

## Methodology and study design

**Fig. 1:**
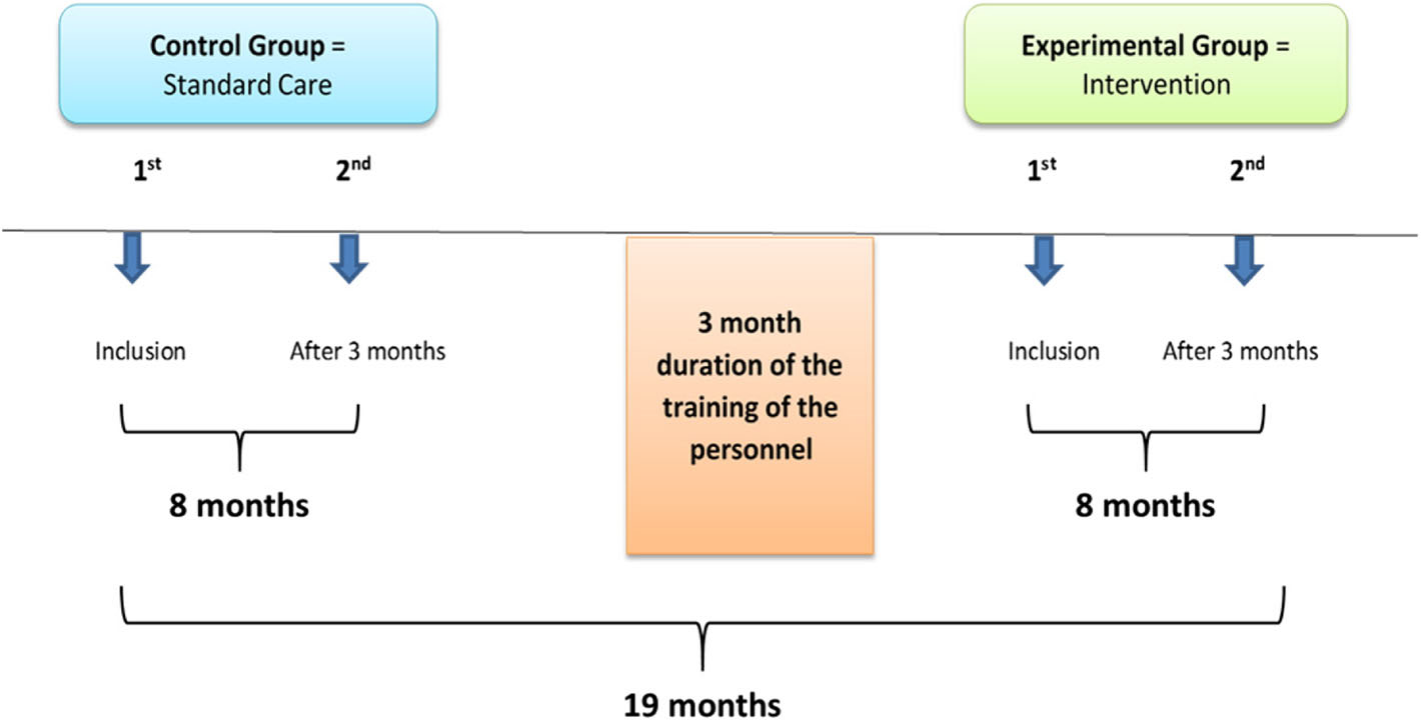
Sequence of the feasibility before-after intervention study

We propose to conduct a before-after intervention study consisting of three sequential phases: baseline phase, training phase, and intervention phase. During the baseline phase, the control group will receive standard care. During the training period, the personnel will be trained on how to use and apply the “early palliative care integration” intervention. Finally, during the intervention phase, the intervention group will receive the updated care (Fig. 1).

In both groups (control and intervention), patients and their most important informal caregiver will be asked to complete the Canadian Health Care Evaluation Project (CANHELP Lite) Questionnaire [28], the Palliative Outcome Scale (POS) Questionnaire [29], and the Advance Care Planning Questionnaire developed by the Palliative Support Team (at the University Hospital Leuven) to measure the current level of integrated PC in the following two timings: (i) inclusion moment after signing the informed consent and (ii) 3 months after inclusion (for both baseline and intervention groups). The methodology of this study was developed based on the SPIRIT guidelines.

We decided to jointly study CHF and COPD due to the fact that these two chronic, high prevalence, and non-malignant diseases share heavy physical and psychosocial symptom burden and similarly complicated disease trajectories, and currently, they are not curable [30, 31]. Moreover, there is also empirical evidence that CHF/COPD specialists share similar views on the role of integrated PC for these two diseases [32].

Currently in our hospital, the acute wards outside oncology have access to the Palliative Support Team, but they typically call for it only in the last hours (in the best case, in the last 2–3 days) of a patient’s life. The personnel of the cardiology and pulmonology wards have no training on the provision of PC.

### Inclusion criteria

#### Patients

Adult patients have been readmitted at least once within a year with a New York Heart Association (NYHA) classification of III/IV for CHF (Table 1) or with Global Initiative for Chronic Obstructive Lung Disease (GOLD) stages C/D for COPD (Table 2). These patients should have an assessed life expectancy of at least 6 months.

**Table 1:** NYHA classification for CHF symptoms

NYHA class	Symptoms
I	Cardiac disease, but no symptoms and no limitation in ordinary physical activity, e.g., no shortness of breath when walking, climbing stairs, etc.
II	Mild symptoms (mild shortness of breath and/or angina) and slight limitation during ordinary activity
III	Marked limitation in activity due to symptoms, even during less-than-ordinary activity, e.g., walking short distances (20–100 m) Comfortable only at rest
IV	Severe limitations, experiences symptoms even while at rest, mostly bed-bound patients

*NYHA* New York Heart Association

**Table 2:** GOLD criteria for COPD

A = low risk, low symptom burden	
• Low symptom burden (mMRC of 0–1 or CAT score < 10) and	
• FEV1 of 50% or greater (old GOLD 1–2) and low exacerbation rate (0–1/year)	
B = low risk, higher symptom burden	
• Higher symptom burden (mMRC of 2 or more or CAT of 10 or more) and	
• FEV1 of 50% or greater (old GOLD 1–2) and low exacerbation rate (0–1/year)	
C = high risk, low symptom burden	
• Low symptom burden (mMRCof 0–1 or CAT score < 10) and	
• FEV1 < 50% (old GOLD 3–4) and/or high exacerbation rate (2 or more/year)	
D = high risk, higher symptom burden	
• Higher symptom burden (mMRC of 2 or more or CAT of 10 or more) and	
• FEV1 < 50% (old GOLD 3–4) and/or high exacerbation rate (2 or more/year)	

*mMRC* Modified Medical Research Council Dyspnea Scale, *CAT* COPD assessment test, *FEV1* the amount of air a patient can force from his/her lungs in 1 s

#### Informal caregivers

Informal caregiver is defined as the proxy person who takes care and supports the patient for most of the time. This caregiver may not necessarily be a family member. They should be aged 18 or above, should be able to communicate in Dutch, and should be cognitively able to complete questionnaires.

### Exclusion criteria

A potential participant (patient or informal caregiver) who meets any of the following criteria will be excluded from participation in the study:

i) Under the age of 18
ii) Unable to communicate in Dutch
iii) People who lack the mental capacity to complete questionnaires
iv) If the surprise question “Would you be surprised if the patient died within 1 year?” is answered “Yes”

### Sample size

A convenience sample of 50 patients and 50 informal caregivers per phase (25 CHF and 25 COPD patients and their informal caregivers for the baseline phase and 25 CHF and 25 COPD patients and their informal caregivers for the intervention phase) has been set as a target based on the following rationale. We use as a reference a two-sample *t* test on the total POS scores, assuming a minimally relevant difference of 4, i.e., 10% of the best score 40, and a common standard deviation of 6, which constitutes the worst case based on previous studies [33, 34]. In order to obtain 80% power at a significance level of 5%, a total of 37 patients per group will be required. To this number, we will add a 30% attrition rate which results in 50 patients and caregivers per group. We will retrospectively compute our standard deviation with an interim analysis once three fourths of the originally planned sample size has been collected to examine whether upward revisions are required.

It is important to note that due to the shortage of studies similar to ours, attrition rates for the population of interest are not well understood. As a starting point, we have used attrition rates from integrated palliative care cancer studies and we aim to retrospectively evaluate the suitability of these rates.

### Duration and setting

The overall study is estimated to last 24 months and will be conducted in UZ Leuven in the Departments of Cardiology and Pulmonology (two nursing wards for CHF and one for COPD). We estimate 8 months duration for the baseline and the intervention phase and 3 months for the training period. An overall time delay of 25% has also been added to the timeline.

### Feasibility assessment

For the feasibility assessment of our study, we have employed the analytical framework of Bugge et al., adapted directly from Kane et al. [35, 36]. The assessment is reported in Table 3 where the researchers have to complete the findings and evidence columns for each of the 14 methodological issues.

**Table 3:** Feasibility assessment of 14 methodological issues

Methodological issues	Findings	Evidence
1. Did the feasibility study allow a sample size calculation for the main trial?		
2. What factors influenced eligibility and what proportion of those approached were eligible?		
3. Was recruitment successful?		
4. Did eligible participants consent?		
5. Were participants successfully randomized and did randomization yield equality in groups?		
6. Were blinding procedures adequate?		
7. Did participants adhere to the intervention?		
8. Was the intervention acceptable to the participants?		
9. Was it possible to calculate intervention costs and duration?		
10. Were outcome assessments completed?		
11. Were outcomes measured those that were the most appropriate outcomes?		
12. Was retention to the study good?		
13. Were the logistics of running a multicenter trial assessed?		
14. Did all components of the protocol work together?		

### Questionnaires

The CANHELP Lite Questionnaire is a tool that is often used in clinical PC studies to evaluate the quality of care [28]. This is a validated questionnaire with two versions: one version for patients and one for the informal caregivers. It can be used in both inpatient and outpatient care settings, and it has a completion time of approximately 10 min. The patient version has 21 questions that address the satisfaction of the quality of care, relationship with the doctors, illness management, communication, decision-making, and a question concerning the patient’s inner peace. For the caregivers, the questionnaire includes 23 questions assessing the satisfaction of the overall care, the relationship with the doctors, the characteristics of doctors and nurses, the illness management, the communication, the decision-making, and the involvement of the informal caregiver to patient’s care.

The POS is a validated tool that is frequently used in PC studies to measure (i) the quality of life and (ii) the symptoms of patients. This questionnaire has two versions, one for patients and one for informal caregivers, and it has a completion time of approximately 7 min [29]. This questionnaire consists of 12 questions, same for both patients and informal caregivers addressing their overall quality of life including physical and emotional symptom management and adequacy of information received.

The Advance Care Planning Questionnaire was developed by the Palliative Support Team of UZ Leuven, and it is used to measure different elements of advance care planning. It has three versions: one for patients, one for the informal caregivers, and one for healthcare providers (in this study, the patient and informal caregiver versions will be used). It has a completion time of approximately 10 min. Due to the fact that currently there exist no questionnaires that measure existing advance care planning aspects, this questionnaire is considered the most suitable for the goal of our study; however, it is yet to be validated. This questionnaire addresses questions over the perceptions of patients and caregivers concerning the current care, usefulness of discussions related to patient’s health, desire for involvement in these discussions in the present and the future, and evaluation of their emotional condition.

### Recruitment

According to the design of the study, and following discussions with the healthcare providers of pulmonology and cardiology wards, the following model for the identification of eligible patients will be adopted. The researcher, in charge after screening the patients’ electronic hospital files and after consulting with the head nurses of the cardiology wards and participating in their weekly multidisciplinary meetings of the wards, will identify the eligible CHF patients. For COPD, a pulmonology ward nurse is appointed to screen and report the eligible patients to the researcher upon examining the electronic files of the patients and cross-checking with assistant physicians of the corresponding ward during bilateral or multidisciplinary meetings. The information of the eligible COPD patients will be communicated via e-mail with the researcher. If any difficulties occur in the implementation of the recruitment process, the research team will perform the necessary adjustments so as to enhance the recruitment process.

### Data collection

The main researcher (NS) will explain the study protocol to both patient that meets the inclusion criteria and his/her most important informal caregiver. The patient will receive the informed consent document. After 1 or 2 days of reflection time, the main researcher will contact them again personally to ask if they like to join the study. If the patient and informal caregiver decide to participate in the study, they will provide their signed consent.

After the completion of the informed consent, the participants (patients and their informal caregiver) will be requested to fill in the three questionnaires in two timings: (1) at inclusion moment after signing the informed consent during the hospitalization of the patient and (2) at a follow-up of 3 months after inclusion (for both control and intervention groups). If a patient or an informal caregiver has difficulty understanding the questionnaires, the researcher in charge can provide additional information and help the participants fill in the questionnaires. When the eligible patients or their informal caregivers decline to participate, the researcher will ask for the reason and archive it. No special document will be provided to patients who will decline. Communication of the reason for not participating will be collected by the recruiter based on oral communication and without a predefined set of options in order to capture a broader spectrum of answers.

**Fig. 2:**
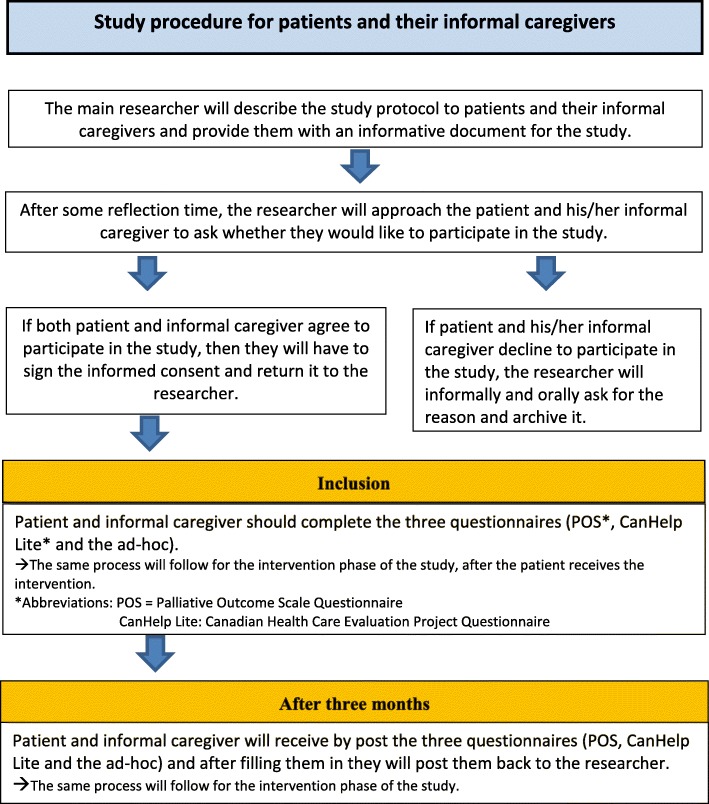
Study procedure for patients and their informal caregivers

For the second measurement after 3 months, the same questionnaires will be sent by post to each patient and their caregiver and will be sent back to the researcher after they are completed. In some cases, the follow-up questionnaires will be administered to the patients when they visit the hospital for a checkup examination. When the follow-up questionnaires that have been posted are not returned back on time, the researchers will then contact the participants by telephone to remind them to complete and post the questionnaires back. The same process will be repeated for each patient and his/her caregiver for the intervention phase and after the implementation of the intervention. The study procedure for patients and their informal caregivers is described in Fig. 2.

### The intervention

After the completion of the baseline phase of the study (patients receiving standard care), the appointed intervention team for each ward will receive a training on how to apply the intervention in a 3-month period. The psychologist of UZ Leuven’s Palliative Support Care Team will be the one to train the personnel on how to apply the intervention to the experimental group. In cardiology, the assistant physicians will provide an introductory flyer for the intervention to the patient, and after the completion of this flyer, two psychologists and one psychology trainee will proceed with the implementation of the intervention. The same process will follow for the pulmonary ward, where after the introductory flyer by the assistant physicians, a specialist nurse, two ward nurses, and a spiritual counselor will proceed with the intervention.

The intervention will be based on the implementation of the early integrated palliative care planning intervention “My Wishes” (“MIJN WENSEN voor mijn Gezondheidszorg”), a content-validated communication tool for advance care planning in chronic disease developed by the Palliative Support Team in UZ Leuven (reference number 700864).

This tool addresses the wishes and needs of the patients concerning the delivery of care. It considers how the patient’s needs may be translated into treatment agreements for the present and the future taking into account the medical possibilities and limitations. MIJN WENSEN will be used as an invitation to communication and as a guide for conversations with the caregivers and for matching the current and future care with the treatment specialists. MIJN WENSEN can be also used as an informal declaration of intent, to register the patient’s indicated representative who will advocate the wishes of the patient in case she/he ever becomes unable of taking any decisions on their medical care. It can even be used as a formal declaration of intent, not only for the indication of a representative, but also the content of the treatment wishes for every type of mental incompetence.

Overall, MIJN WENSEN covers several components of the integrated PC like advance care planning, discussions on prognosis and illness limitations, provision of a holistic approach (physical, psychosocial, spiritual), patients’ goal assessment and continuous goal adjustment, financial issue discussions, DNR codes, (un)desirable treatments for current and future care, patients’ wishes, possibility of involvement of a PC team, and care for the last hours of life. Some components like the bereavement care and the early and direct involvement of a PC team are not included.

#### Data analysis

The statistical analysis will be carried out by the main researcher and will be performed using SPSS version 23.0 and Excel version 18.11 software. We will employ standard descriptive statistics for the basic characteristics of the population and the distributions of the scores of the tests (means, standard deviations, etc.). We will use *t* tests to explore the equality of means for total scores of POS and CANHELP Lite between different pairs (e.g., COPD patients vs COPD caregivers). To compare continuous variables between the different study groups, we will use Fisher’s exact tests and Mann-Whitney *U* test. Finally, we will use Mann-Whitney *U* tests and Wilcoxon signed-rank tests in order to analyze individual questions that will be treated as ordinal variables, while 95% confidence intervals will also be presented. Missing data will be included in the analysis, and their implications will be clarified.

#### Expected results

The present study will first evaluate the feasibility of carrying out PC-focused studies in acute wards where PC is not considered as part of the treatment teams. This will allow us to detect possible weaknesses and strengths by observing the real-time interaction of the medical staff with the patients. The learned lessons can then be juxtaposed with existing evidence and thus lead to recommendations and suggestions for the improvement of guidelines for the implementation of PC services outside PC wards.

Second, by evaluating current practices, the study will document the state of the art from the viewpoint of CHF/COPD patients. Since the needs of this population are not well understood, these results can help towards the development of PC practices tailored to the needs of these patients.

Third, the study will measure the potential efficacy of the intervention against existing practices. By doing so, we will be able to quantify the possible benefits of the integrated PC in CHF/COPD.

Fourth, our study constitutes the first and minimally invasive step towards integrating PC in these acute wards. The continuous presence of an integrated PC research team will foster interactions with all the members of the two acute settings and help shifting the current mentality.

Finally, through the application of the “early integrated palliative care” intervention, the medical staff will be able to promptly identify the PC needs of CHF/COPD patients and their caregivers while being able to address them in a more efficient manner thereby improving the existing quality of care and quality of life of these patients.

## Discussion

### Ethical considerations

The process of filling in the questionnaires could be potentially stressful for the patients and their informal caregivers. In order to minimize the risk of emotional distress, we have chosen for this study questionnaires that have a short completion time (based on previous empirical studies completion time for POS is approximately 7 min and for CANHELP Lite is 10 min). The overall risk of the study including societal risks or risks related to the design and the performance is imperceptible.

The advantage of this study is that both patients and their informal caregivers will be given the chance to share their opinion for their chronic illness and to express their preferences for their care. Even though the patients and their informal caregivers might not be directly affected from the study, providing their feedback can have a significant impact for the improvement of the quality of care of such patients in the future.

### Informed consent

The main researcher will explain the study protocol to both the patient who meets the inclusion criteria and his/her most important informal caregiver. The patient and the informal caregiver will receive the informed consent and a letter with the description of the study. After 1 or 2 days of reflection time, the researcher (NS) will contact them again personally to ask if they like to join the study, which means that (a) they will have the patient’s electronic records searched and (b) both patients and informal caregivers will be asked to fill in two questionnaires at inclusion and 3 months after inclusion. If the patient and informal caregiver decide to participate in the study, they will provide a signed consent. If there is no informal caregiver or the informal caregiver does not want to participate, then we will still include the patient in the study.

### Data processing

All the data retrieved from the electronic files of the patients and all the questionnaires filled in by the patients and their informal caregivers will be treated with strict confidentiality and will not be made public. The findings of the study will be processed following the regulations of the “Belgian law on data protection” (Belgische wet op gegevensbescherming).

After the collection of the data and the analysis, the main researcher will write the manuscripts and submit them to international PC-related peer-reviewed journals. Publications will be coordinated by the principal investigator of the project. The authorship of the publications will be regulated according to the requirements of the International Committee of Medical Journal Editors and according to the requirements of the respective targeted journals.

### Post-feasibility study planning

The long-term objective concerns the integration of early PC in the disease trajectory of CHF and COPD patients in UZ Leuven. This feasibility study constitutes the first step in this direction. Following its completion, the researchers and the personnel will engage into a series of workshops discussing the outcomes and the lessons learned. The outcomes of these workshops will be translated into practical guidelines for the further integration of PC in CHF/COPD in UZ Leuven. If this feasibility study shows encouraging outcomes for CHF/COPD patients and their informal caregivers, the next step would be to involve cardiology and pulmonology wards from other hospital and conduct an RCT study with the same objectives.

## Limitations

A limitation of this study is its design since we have decided against an RCT. We have extensively discussed the possibility for administering an RCT instead of a before-after study with all involved actors. However, the major obstacle for doing so was the fact that in both cardiology wards and pulmonology wards, there was a substantial overlapping of the intervention-involved personnel (psychologists, pastoral consultants). It was impossible to remove this bias given the organizational constraints of the wards, and for this reason, we decided that a before-after design would more controllable. The final report of our study will include recommendations for future RCT studies and guidelines on how to circumvent the emerging challenges.

Another limitation of this study is the use of the self-developed Advance Care Planning Questionnaire that has not yet been validated and published. This fact will be taken into consideration when performing the statistical analysis.

## Current status of the study

The present study received ethics clearance in March 2016. This was followed by a period of discussions and study planning with the involved wards, and the study is currently ongoing. Its results as well as the lessons learned will be communicated in a series of future publications.

## Abbreviations

CANHELP: Canadian Health Care Evaluation Project
CHF: Chronic heart failure
COPD: Chronic obstructive pulmonary disease
GOLD: Global Initiative for Chronic Obstructive Lung Disease
NYHA: New York Heart Association
PC: Palliative care
POS: Palliative Outcome Scale
